# Dihydroartemisinin ameliorates psoriatic skin inflammation and its relapse by diminishing CD8^+^ T-cell memory in wild-type and humanized mice

**DOI:** 10.7150/thno.45211

**Published:** 2020-08-21

**Authors:** Yuchao Chen, Yuhong Yan, Huazhen Liu, Feifei Qiu, Chun-Ling Liang, Qunfang Zhang, Run-Yue Huang, Ling Han, Chuanjian Lu, Zhenhua Dai

**Affiliations:** 1 State Key Laboratory of Dampness Syndrome of Chinese Medicine, the Second Affiliated Hospital of Guangzhou University of Chinese Medicine, Guangzhou, Guangdong 510120, China.; 2 Section of Immunology and Joint Immunology Program, Guangdong Provincial Academy of Chinese Medical Sciences, Guangzhou, Guangdong 510006, China.

**Keywords:** Dihydroartemisinin, Immunosuppression, Psoriasis, Relapse, Memory T cells

## Abstract

Conventional immunosuppressants cause side effects and do not prevent the recurrence of autoimmune diseases. Moreover, they may not inhibit autoimmunity mediated by pathogenic memory T-cells. Dihydroartemisinin (DHA) has been shown to regulate autoimmunity. However, it remains unknown whether DHA impacts psoriasis and its recurrence. The objective of this study was to determine therapeutic effects of DHA on psoriasis and its relapse as well as its underlying mechanisms.

**Methods:** We established animal models of imiquimod (IMQ)-induced psoriasis-like wild-type mice and humanized NSG mice receiving lesional human skin from patients with psoriasis. Many immunoassays, including immunohistochemistry, flow cytometry, quantitative RT-PCR and Western blotting, were performed.

**Results:** We found that DHA not only ameliorated acute skin lesion of psoriatic mice, but also alleviated its recurrence by diminishing CD8^+^ central memory T (T_CM_) and CD8^+^ resident memory T (T_RM_) cells. It attenuated epidermal pathology and T-cell infiltration in the skin of IMQ-induced psoriatic mice while suppressing expression of IL-15, IL-17 and other proinflammatory cytokines in the skin. Surprisingly, DHA reduced the frequency and number of CD8^+^, but not CD4^+^, subset of CD44^high^CD62L^high^ T_CM_ in psoriatic mice, whereas methotrexate (MTX) lowered CD4^+^, but not CD8^+^, T_CM_ frequency and number. Indeed, DHA, but not MTX, downregulated eomesodermin (EOMES) and BCL-6 expression in CD8^+^ T-cells. Furthermore, DHA, but not MTX, reduced the presence of CD8^+^CLA^+^, CD8^+^CD69^+^ or CD8^+^CD103^+^ T_RM_ cells in mouse skin. Interestingly, treatment with DHA, but not MTX, during the first onset of psoriasis largely prevented psoriasis relapse induced by low doses of IMQ two weeks later. Administration of recombinant IL-15 or CD8^+^, but not CD4^+^, T_CM_ cells resulted in complete recurrence of psoriasis in mice previously treated with DHA. Finally, we demonstrated that DHA alleviated psoriatic human skin lesions in humanized NSG mice grafted with lesional skin from psoriatic patients while reducing human CD8^+^ T_CM_ and CD103^+^ T_RM_ cells in humanized mice.

**Conclusion:** We have provided the first evidence that DHA is advantageous over MTX in preventing psoriasis relapse by reducing memory CD8^+^ T-cells.

## Introduction

Psoriasis is an immune-mediated chronic skin disease, which is prone to relapse and affects approximately 2% of the population worldwide 1. Although the pathogenesis of psoriasis remains unclear, it has been widely recognized that T cells, especially CD4^+^ Th17 and IL-17-producing γδ T cells, play an important role in the development and progression of psoriasis 2, 3 and cutaneous DTH responses 4. The accumulation of Th17 cells in the dermis can lead to the aberrant differentiation and proliferation of keratinocytes by producing abundant proinflammatory cytokines, such as IL-6, IL-17A and IL-22 5. Interestingly, recent findings have demonstrated that CD8^+^ T cells can also mediate psoriasiform skin inflammation 6. More recent studies have highlighted an important role for memory T cells in the pathogenesis and manifestation of psoriasis, especially its recurrence 7-9. It has been shown that either CD4^+^ or CD8^+^ central memory T (T_CM_) cells are increased in circulating peripheral blood of psoriasis patients 10, 11. T_CM_ cells can also expand upon re-challenging *in vitro* and develop into Th17 cells 12. On the other hand, resident T or resident memory T (T_RM_) cells persist for long term in the skin and do not recirculate through the blood 13, 14. Previous studies have shown that T_RM_ cells are enriched in both active and resolved psoriatic skin lesions 15, 16. They can also cause the recurrence of skin lesion in the same region by producing IL-17 16, 17. Although T_RM_ cells may include both CD4^+^ and CD8^+^ subsets 18, skin CD8^+^ T_RM_ cells expressing CD69, CD103 and CLA have been recently revealed in the context of psoriasis 17, 19. Therefore, targeting memory T cells, especially CD8^+^ T_RM_, may be a promising approach to treating psoriasis and its recurrence.

Conventional immunosuppressive agents, including cyclosporine A, methotrexate (MTX), acitretin and apremilast, are available for treating psoriasis. However, substantial side effects of these drugs have been observed 20, 21. On the other hand, few psoriatic patients receive treatment with biologics because of their high cost, leading to limitation of their application in clinic 22. Skin lesions recur in many patients with psoriasis after they stop taking the biologics. Therefore, it is compelling to explore new drugs with potentially low cost, less side effects and low recurrence rate for psoriasis treatment.

Artemisinin, an active ingredient isolated from Chinese herb *Artemisia annua* L*.*, has been used to treat malaria for decades 23. Chinese medicine has been shown to be effective in treating psoriasis 24. Tu Youyou was awarded the Nobel Prize in Physiology or Medicine in 2015 for her discovery of the anti-malarial effects of artemisinin 25, 26. However, because of poor solubility and short half-life of artemisinin, researchers have developed its derivatives that exhibit better bioavailability. Dihydroartemisinin (DHA), a derivative of artemisinin, has better bioavailability and anti-malarial effects than artemisinin 27. Recently, DHA has been shown to exert anti-fibrosis 28, 29 and anti-inflammatory effects 30, 31. In particular, DHA has been demonstrated to mainly regulate autoimmunity in several animal models of autoimmune diseases, including autoimmune thyroiditis, experimental autoimmune encephalomyelitis, collagen-induced arthritis and ovalbumin-induced allergic asthma 32-35. Tu Youyou's research team has also confirmed that DHA exerts therapeutic effects on lupus nephritis in animal models 36, 37. Although the mechanisms by which DHA ameliorates autoimmune diseases remain largely unknown, they mainly include its suppression of oxidative stress and NF-κB inflammatory pathway and regulation of Th17/Treg balance via modulating mTOR signaling 27-32. These findings indicate that DHA could be an effective drug for the treatment of psoriasis.

In this study, we determined the effects of DHA on imiquimod (IMQ)-induced psoriasiform skin inflammation in WT mice or NSG mice grafted with psoriatic human skin and its underlying mechanisms. We found that DHA significantly ameliorated the psoriasiform skin lesion, inhibited epidermal hyperplasia and reduced T cell infiltration in the skin of the psoriatic mice. It also suppressed mRNA expression of proinflammatory cytokines, including IL-15 and IL-17A, in the psoriatic skin. Importantly, we found that early treatment with DHA, but not MTX, largely prevented the recurrence of the skin lesion in psoriatic mice. This effect was attributed to its downregulation of CD8^+^ T_CM_ and T_RM_ cells in the psoriatic mice. Finally, we demonstrated that DHA not only attenuated human skin lesion in humanized NSG mice grafted with lesional skin from psoriatic patients, but also shrank human CD8^+^ T_CM_ and T_RM_ pool in the humanized mice, indicating its translational value.

## Methods

### Reagents

IMQ cream (Containing 5% IMQ) was purchased from Sichuan Mingxin Pharmaceutical Co., Ltd. (Sichuan, China). DHA (purity ≥ 98%) was obtained from Nanjing Dosifu Biotechnology Co., Ltd. (Nanjing, China). Recombinant Murine IL-15 was bought from PeproTech (USA) while MTX was purchased from SPH Sine Pharmaceutical Laboratories Co., Ltd (Shanghai, China).

### Animals

BALB/c mice (male, 6-8 weeks old, 20 ± 2 g) were purchased from Guangdong Medical Laboratory Animal Center (Guangzhou, China). NSG mice (male, 6-8 weeks old, 20 ± 2 g) were purchased from Nanjing Biomedical Research Institute of Nanjing University (Nanjing, China). All mice were housed under a pathogen-free condition and provided free access to food and water. All animal experiments were carried out in accordance with the National Institutes of Health guide for the care and use of laboratory animals (NIH Publications No. 8023, revised 1978), and approved by the Animal Ethics Committee of Guangdong Provincial Academy of Chinese Medical Sciences.

### IMQ-induced psoriasis-like mouse model and treatment

BALB/c mice were randomly divided into five groups, including control, vehicle, DHA low-dose (DHA-L), DHA high-dose (DHA-H) and MTX. The back hair of the mice was shaved and all mice except control group were topically treated with 62.5 mg IMQ cream on the back skin for 7 consecutive days, as described previously 38. DHA was prepared by a solution containing 1% Tween-80 and 10% PEG400. The mice of DHA-treated groups were intraperitoneally administered with DHA at a dose of 25 (DHA-L) or 50 (DHA-H) mg/kg/day for 7 consecutive days. For comparison, MTX was dissolved in the saline. The mice of MTX group were intraperitoneally administered with MTX at a dose of 1.5mg/kg/day for 7 consecutive days. The mice of control and vehicle groups were only given the solvent (Containing 1% Tween-80 and 10% PEG400) daily for 7 consecutive days. The body weight and PASI scores of the mice were recorded on the first day of IMQ treatment for 7 consecutive days. Mice were sacrificed on day 7 and their blood, skin, spleen and lymph nodes were collected for further analyses.

To observe the recurrence of psoriasis, BALB/c mice were treated with vehicle, DHA (25 or 50 mg/kg) or MTX (1.5 mg/kg) for 7 consecutive days for the first application of 62.5 mg IMQ. After 14 days of recovery, all mice were challenged with secondary application of much smaller doses of IMQ (20.8 mg) for 7 more consecutive days (Figure 4A). The skin lesions and PASI scores were then recorded. To reconstitute T_CM_ cells in DHA-treated mice, mice were injected with exogenous CD4^+^ or CD8^+^ T_CM_ cells (1×10^6^) on day 20 while others received rIL-15 (0.1 μg/each) on days 0, 3 and 6. These T_CM_ cells were isolated from mice treated with IMQ but without DHA on day 20.

### Scoring the severity of murine psoriatic skin lesion

The severity of murine psoriatic skin lesion was evaluated according to Psoriasis Area and Severity Index (PASI), which was modified from a scoring system of human psoriasis area and severity index. The modified PASI has three parameters, including skin erythema, scales and thickness. Three parameters were scored independently from “0” to “4”. “0” represents none; “1” represents slight; “2” represents moderate; “3” represents marked; “4” represents very marked. The specific scoring criteria were described previously 39.

### Histological analysis and immunohistochemistry (IHC)

Skin samples from mice were fixed in 4% neutral paraformaldehyde for 24 h and then embedded in paraffin. The skin samples in paraffin were cut into 3 μm-thick sections and placed on slides. The skin sections were then stained with hematoxylin and eosin (H&E staining). To measure acanthosis, the epidermal area was outlined, and its pixel size was measured. The relative area of the epidermis was calculated using the formula as follows: area=pixels/ (horizontal resolution × vertical resolution). The papillomatosis index was typically measured as previously reported 13. For IHC staining, skin sections were heat-mediated using citric acid buffer (pH 6.0) for 5 to 8 min followed by cooling at room temperature for 20 min. Then, skin sections were incubated with primary anti-Ki67 (ab16667, 1:100) or anti-CD3 (ab16669, 1:100) monoclonal antibody (Abcam, Cambridge, UK) at 4 °C overnight. HPR-conjugated goat anti-rabbit IgG (Maxim, China) was used as the secondary antibody at room temperature for 30 min. Finally, the sections were stained with diaminobenzidene (DAB, Sigma-Aldrich) and counterstained by hematoxylin. For quantitative analysis, the number of Ki67^+^ cells and the integrated optical density (IOD) of CD3 were measured using ImagePro Plus 6 software. For immunofluorescence staining, the skin sections were incubated with anti-CD103 antibody (ab224202, 1:100) at 4 °C overnight. Sections were then incubated with Alexa Fluor^®^ 488-conjugated goat-anti rabbit IgG (ab150081, 1:500) at room temperature for 1 h. Finally, sections were mounted by DAPI Fluoromount-G^®^ (SouthernBiotech, Birmingham, UK). The fluorescence intensity of CD103 was also measured using the ImagePro software.

### RNA extraction and reverse transcription quantitative PCR (RT-qPCR)

Total RNA was extracted from murine skin tissues with TRIzol reagents (Invitrogen, USA). RNA was transcribed to cDNA using PrimeScript^TM^ RT reagent kit (Takara Bio Incorporation, Kusatsu, Japan) according to the instruction of the manufacturer. Quantitative PCR was then performed on ViiA 7 Dx (Applied Biosystems) using SYBR Premix Ex Taq^TM^ Π (Takara Bio Incorporation). GAPDH gene was used as an internal standard gene and the 2^-ΔΔCT^ method was utilized to quantitatively analyze the data. The primer sequences are shown below: IL-6 Forward: ACTTCCATCCAGTTGCCTTCTTGG, Reverse: TTAAGCCTCCGACTTGTGAAGTGG; IL-15 Forward: ACATCCATCTCGTGCTACTTGT, Reverse: GCCTCTGTTTTAGGGAGACCT; TNF-α Forward: ACGGCATGGATCTCAAAGAC, Reverse: GTGGGTGAGGAGCACGTAGT; IFN-γ Forward: CACGGCACAGTCATTGAAAG, Reverse: CATCCTTTTGCCAGTTCCTC; IL-17A Forward: GTCCAAACACTGAGGCCAAG, Reverse: ACGTGGAACGGTTGAGGTAG; GAPDH Forward: AGGTCGGTGTGAACGGATTTG, Reverse: TGTAGACCATGTAGTTGAGGTCA.

### Western blotting

Total protein samples from skin tissues were obtained using RIPA lysis buffer followed by centrifugation at 12,000 g for 10 min. The concentration of proteins in the supernatant was measured using a BCA protein assay kit (Thermo Fisher Scientific). Then protein samples were run in 10% SDS-PAGE gel and transferred to PVDF membranes. The membranes were blocked with TBST containing 5% (w/v) BSA at room temperature for 1 hour and then incubated with primary anti-phospho-p65, anti-p65, anti-phospho-IKKα, and anti-IKKα antibodies (1:1,000, Cell Signaling Technology, Boston, USA) at 4 °C overnight. After incubation, the membranes were washed using TBST and incubated with HPR-conjugated goat anti-rabbit or anti-mouse IgG (1:2,000) at room temperature for 1 hour. Blots were detected by a Bio-Rad Gel imaging system and analyzed using Image Lab software.

### ELISA analysis

The total protein from skin was obtained as described above. The levels of IL-15Rα in the skin were detected using mouse IL-15Rα ELISA kit according to the manufacturer's instructions (Boster, China). The serum was obtained from blood by centrifugation at 3,000 rpm for 15 min. The levels of IL-17A, IL-15, IFN-γ and TNF-α in the serum were also detected using the corresponding ELISA kits (Boster, China).

### Flow cytometric analysis

**Figure 1:**
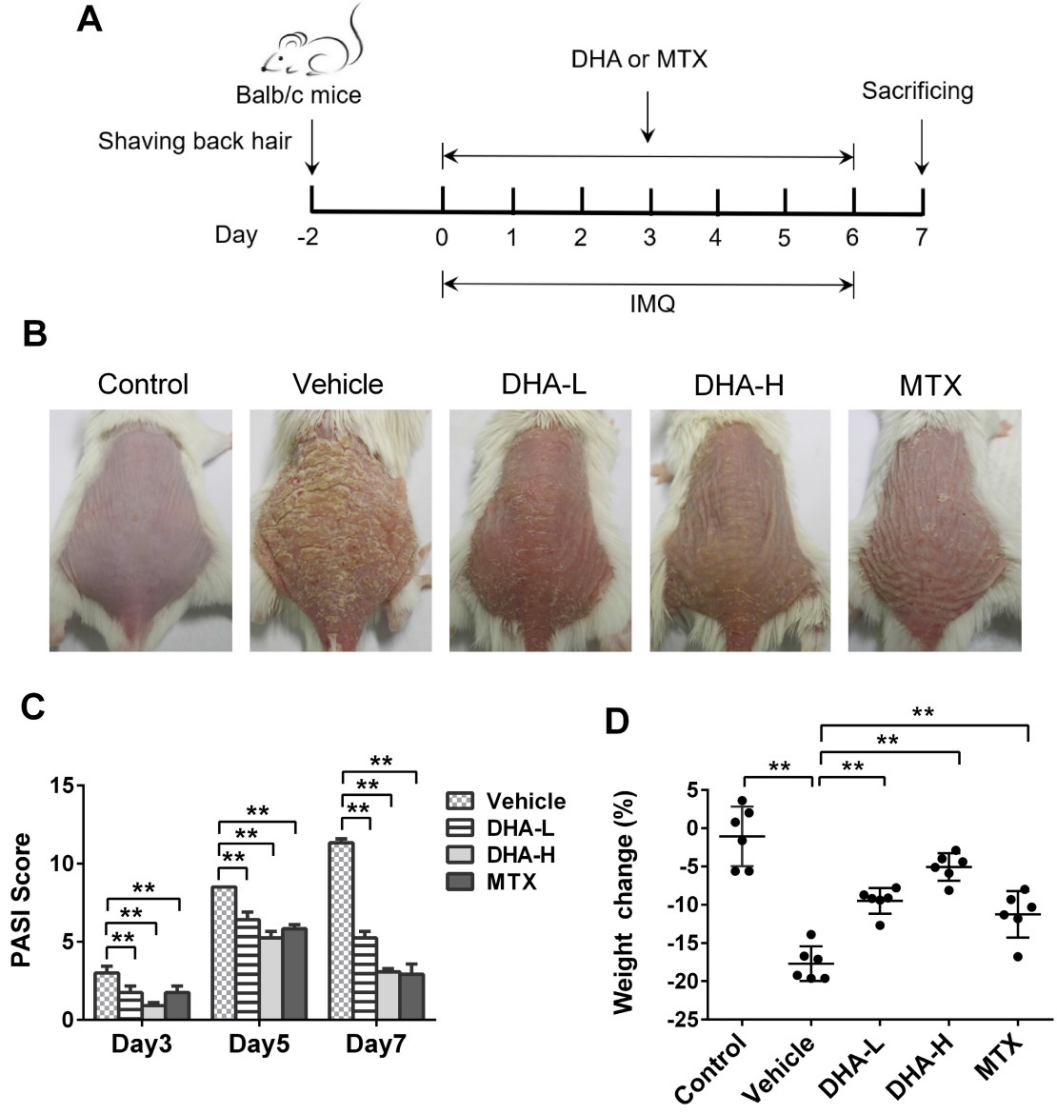
** DHA reduces the PASI scores and ameliorates the skin lesion of IMQ-induced psoriasis-like mice.** (**A**) Shown is a timeline of various treatments. BALB/c mice were intraperitoneally administered with DHA (DHA-L: 25 mg/kg; DHA-H: 50 mg/kg) or MTX (1.5 mg/kg) for 7 consecutive days during the topical application of IMQ on the dorsal skin. All mice were sacrificed on day 7. (**B**) The representative photos of dorsal skin in mice on day 7. (**C**) The PASI scores of the skin lesion in psoriatic mice on days 3, 5 and 7. (**D**) The body weight changes of the mice on day 7. All data are presented as the mean ± SD from three separate experiments (n = 6 mice/group, *p < 0.05 and **p < 0.01).

Murine draining lymph node and spleen cells were collected and stained for surface markers with anti-CD4-FITC (Clone H129.19) or separately, anti-CD8-FITC (Clone 53-6.7), anti-CD44-PerCP-Cy5.5 (Clone IM7) and anti-CD62L-APC (Clone MEL-14) antibodies (eBioscience or BD Biosciences). Then, cells were fixed and permeated using Fixation/ Permeabilization kits (eBioscience), stained for intracellular markers with anti-EOMES-PE-Cy7 (Clone Dan11mag) and anti-BCL-6-PE (BCL-DWN) antibodies (eBioscience), and finally analyzed using a flow cytometer (FACSAriaⅢ, BD Biosciences). The gating strategy for spleen and LN cells were included in the supplementary material (Figure 1S). To isolate skin-infiltrating cells, murine skin samples were cut into 3-4 mm pieces and washed with HBSS containing calcium and magnesium. Samples were then digested with 200 U/mL Collagenase Type I (Life Technologies) and 100 μg/mL DNAse (Roche) in HBSS (supplemented with 10% FBS) at 37 °C for 4 h. A single-cell suspension was obtained by filtering through a 40-μm cell strainer. Cells were then stained with Live/Dead Violet Kit (Invitrogen) to exclude dead cells, followed by anti-CD8-FITC (Clone 53-6.7), anti-CD69-PE (Clone H1.2F3), anti-CD103-PE-Cy7 (Clone 2E7) and anti-CLA-PerCP-Cy5.5 (Clone HECA-452) mAbs (eBioscience or Biolegend). The stained cells were also analyzed *via* FACS. To detect human T_CM_ cells, draining lymph node cells were isolated from humanized NSG mice that received both PBMCs and lesional skin grafts from psoriasis patients. Cells were then stained with anti-human CD4-eFluor 450 (Clone RPA-T4), CD8a-PE-Cy7 (Clone RPA-T8), CD45RO-APC (Clone UCHL1) and CCR7-PE (Clone 3D12), and analyzed by FACS.

### Collection of skin and blood samples of psoriatic patients

The lesional or non-lesional skin was obtained from psoriatic patients who had no medication for 10 to 20 days, and we were unable to obtain the skin biopsy from psoriasis patients who had no medication for at least 4 weeks, an ideal point as described by others 13. Their PASI scores were 11.0, 4.7, 3.5 and 3.4, respectively, at the time of the skin biopsy. In each experiment, the lesional skin from the same psoriatic patient was used as skin grafts for both untreated and DHA-treated recipient mice. Skin samples were stored in HBSS at 4 ºC once removed. Peripheral venous blood was also taken from the same patients. The protocols of this study using the human samples were performed in accordance with the Declaration of Helsinki Principles and approved by the Ethics Committee on Human Study of Guangdong Provincial Hospital of Chinese Medicine, Guangzhou, China (Approval No: ZF2019-193-01, date: 9/26/2019).

### Psoriatic human skin grafting in humanized NSG mice

Skin recipients were six to eight weeks old NSG mice. After anesthetized, NSG mice were transplanted with non-lesional as control or lesional skin on right dorsal flank area and secured with a sterile bandage. The bandage was removed for observation on day 8 after transplantation. Peripheral blood mononuclear cells (PBMCs) were isolated from blood samples of psoriatic patients by Ficoll centrifugation. 5×10^6^ PBMCs in saline were intravenously injected to the transplanted mice. Each recipient received PBMCs and a skin graft from the same patient. The transplanted mice were divided into non-lesional skin, lesional skin, and lesional skin plus DHA groups. DHA was used at a dose of 50 mg/kg/day upon transplantation. GVHD was not observed within 10 days following PBMC injection. NSG mice were sacrificed on day 8, and the lesional and non-lesional human skin grafts were collected and subject to H&E and immunofluorescence stainings.

### Statistical analysis

Data were presented as the mean ± SD and analyzed using software SPSS version 20. Statistical comparisons between groups were performed using Student's *t*-test and one-way ANOVA. A value of *P* < 0.05 was considered statistically significant.

## Results

### DHA ameliorates the skin lesions of IMQ-induced psoriatic mice

To evaluate whether DHA has an effect on psoriasis, we utilized a model of IMQ-induced psoriatic mice that were treated with DHA, as described in Figure 1A. MTX was initially used as a control for positive results. The representative images of dorsal skin lesions in all groups were shown in Figure 1B. The control mice without IMQ treatment did not show any sign of skin inflammation. However, the mice of vehicle group treated with IMQ had severe symptoms of psoriasis-like skin lesions, including skin erythema, scales and thickness after 7 days of IMQ-treatment. Compared to vehicle, treatments with both low-dose of DHA (DHA-L, 25 mg/kg) and high-dose of DHA (DHA-H, 50 mg/kg) attenuated the skin lesions of psoriatic mice, and so did MTX. Moreover, The PASI scores of DHA-treated or MTX-treated mice were significantly decreased compared with those of vehicle group on days 3, 5, and 7, respectively (Figure 1C). DHA also improved the weight loss in psoriatic mice on day 7 (Figure 1D). Thus, our data suggest that DHA ameliorates the skin lesions of IMQ-induced psoriatic mice.

### DHA inhibits epidermal hyperplasia and T cell infiltration in the skin of psoriatic mice

**Figure 2:**
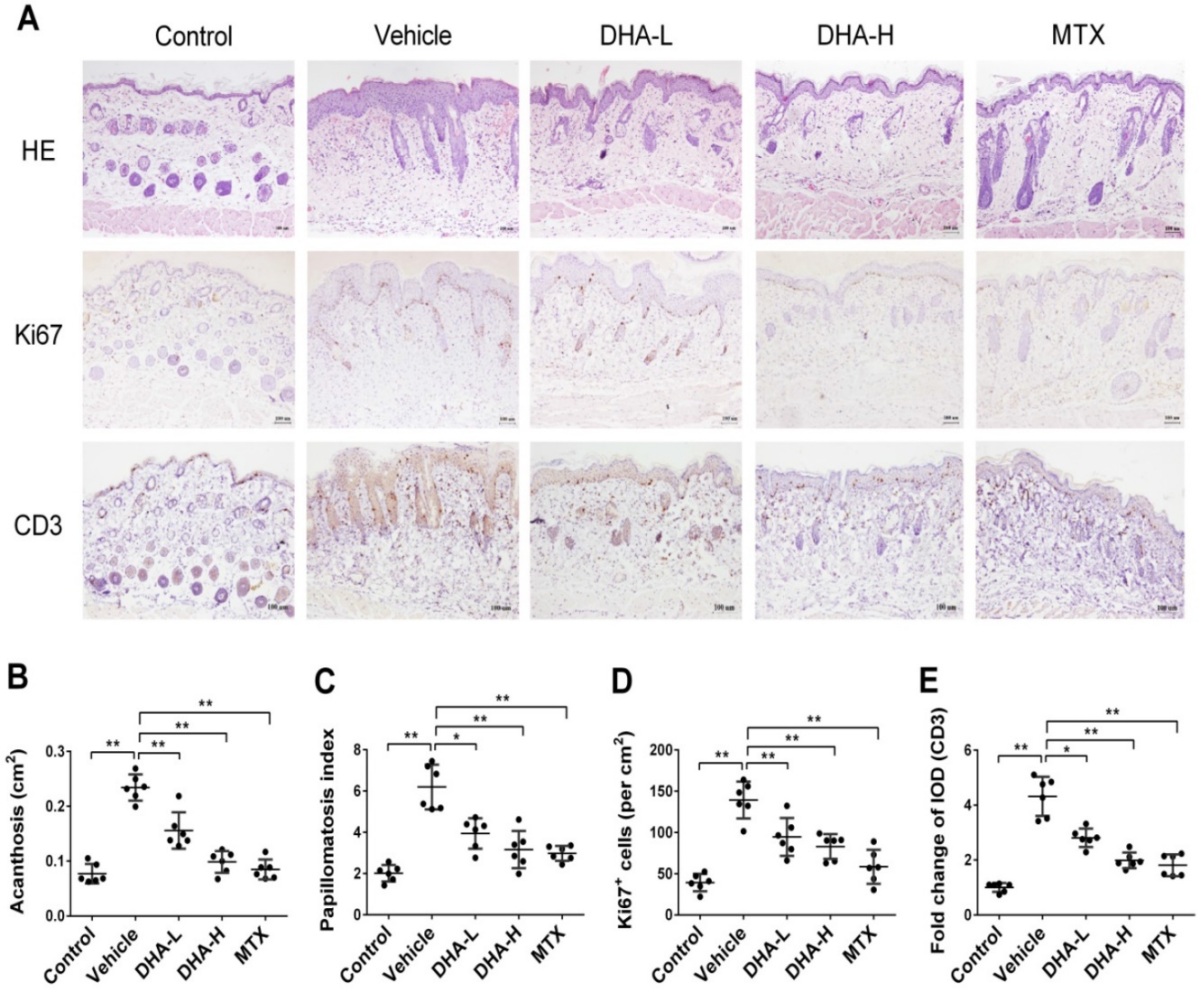
** DHA inhibits epidermal hyperplasia and T cell infiltration in the skin of psoriatic mice**. (**A**) H&E and IHC staining of Ki67 or CD3 on psoriatic mouse skin at Day 7. Representative images of skin sections are presented (Scale bar, 100 µm). (**B-C**) Acanthosis and papillomatosis index of murine skin based on H&E staining. (**D**) Quantitation of Ki67^+^ cells in epidermis. For counting Ki67^+^ cells, six high-power fields in each section of each sample were calculated. (**E**) The integrated optical density (IOD) of CD3^+^ T cells in the skin was calculated using ImagePro Plus. Values were expressed as fold change relative to control group that was set as 1.0. All data are presented as the mean ± SD from three separate experiments (n = 6 mice/group, *p < 0.05 and **p < 0.01).

Epidermal hyperplasia is one of the typical pathological characteristics of psoriasis. H&E staining showed that mice in vehicle group, after 7 days of IMQ treatment, exhibited significant epidermal hyperplasia, acanthosis and papillomatosis, which were alleviated when mice were treated with DHA or MTX (Figure 2A-C). Ki67 is a nucleoprotein involved in cell proliferation. As shown in Figure 2A & D, Ki67 expression in the epidermis of vehicle group was much higher than that of normal control mice. However, its expression in DHA-treated or MTX-treated mice was significantly decreased compared to that in vehicle group, suggesting that DHA inhibits the epidermal hyperplasia in psoriatic mice. Further, IHC staining of CD3 in the skin showed that T cell frequency in the skin of vehicle group was significantly higher than that of control group (Figure 2A). However, an obvious decrease in CD3 expression was observed in mice treated with either DHA or MTX (Figure 2A & E). These results suggest that DHA inhibits T cell infiltration in the lesional skin of psoriatic mice.

### DHA suppresses expression of IL-15 and proinflammatory cytokines as well as NFκB signaling in psoriatic mouse skin

**Figure 3:**
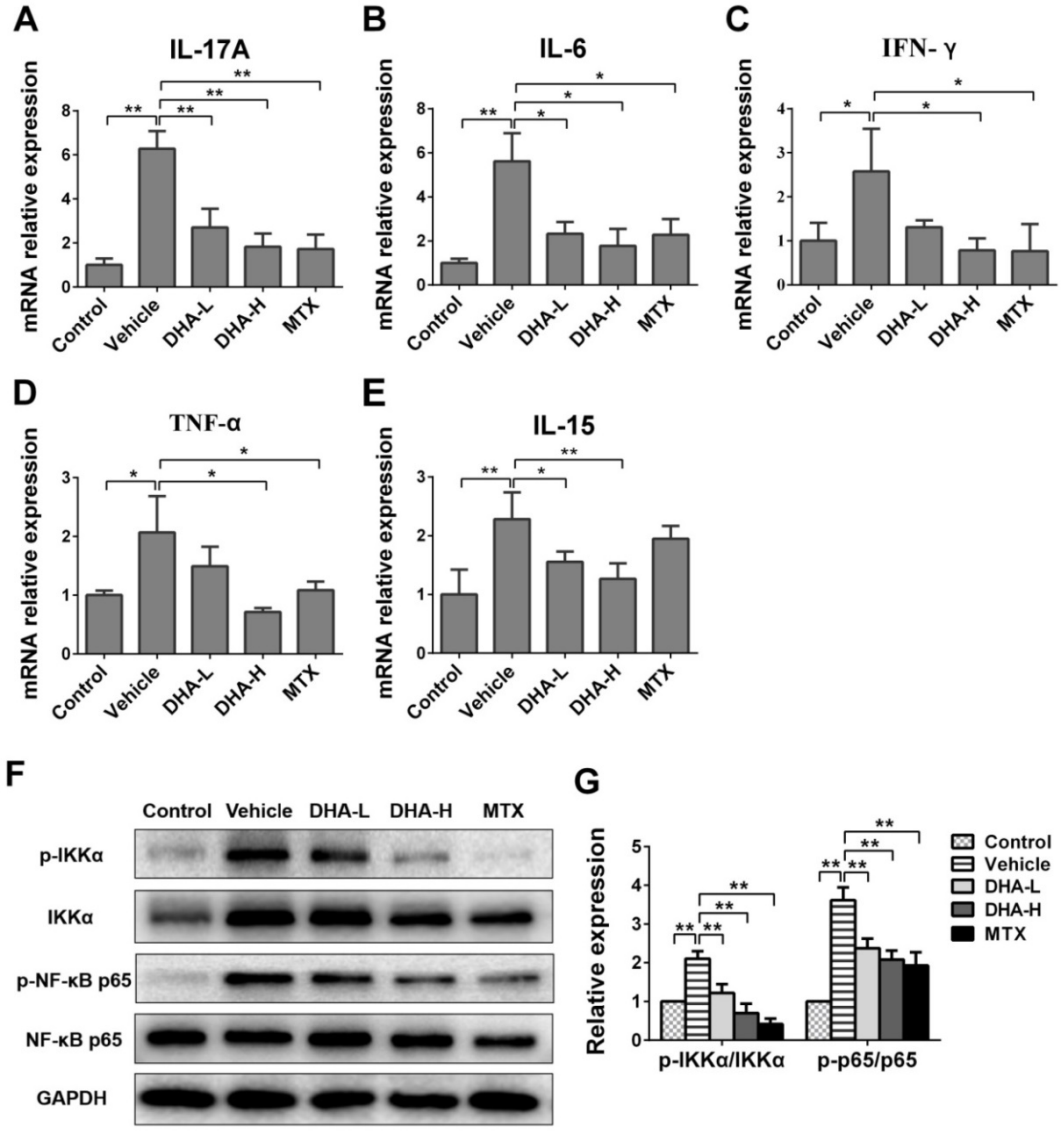
** DHA inhibits mRNA expression of pro-inflammatory cytokines and IL-15 as well as NFκB signaling in psoriatic mouse skin.** The mRNA levels of IL-17A (**A**), IL-6 (**B**), IFN-γ (**C**), TNF-α (**D**) and IL-15 (**E**) in the skin were determined by RT-PCR on Day 7. (**F**) The representative western blots of p-IKKα, IKKα, p-NFκB p65 and NFκB p65 expression in the skin of mice on Day 7. (**G**) Quantification of the relative expression of p-IKKα/IKKα and p-NFκB p65/NFκB p65. GAPDH expression was used to normalize data. Values were expressed as fold changes relative to control group that was set as 1.0. Data of column graphs are presented as the mean ± SD from three separate experiments (n = 4-6 mice/group, *p < 0.05 and **p < 0.01).

To further examine the effects of DHA on expression of proinflammatory cytokines, their mRNA expression in the skin of psoriatic mice was determined by RT-PCR after 7 days of different treatments. As shown in Figure 3A-D, mRNA level of IL-17A, IL-6, IFN-γ or TNF-α in IMQ-treated mice was significantly higher than that in control group. We measured these cytokines since they are important triggers of inflammatory diseases 40-42. As expected, DHA, especially with high doses, reduced mRNA expression of these four proinflammatory cytokines in the skin of psoriatic mice, and so did MTX. But unexpectedly, DHA also significantly inhibited IL-15 mRNA expression in the lesional skin of psoriatic mice (Figure 3E) while MTX failed to do so. Furthermore, DHA lowered protein levels of the cytokines, including IL-15, IL-17A, IFN-γ and TNF-α, in the serum of psoriatic mice while MTX did not reduce IL-15 level (Supplementary material, Figure 2S). However, IL-15Rα level in the skin did not significantly change among all groups, suggesting that neither DHA nor MTX alters IL-15Rα expression (Figure 3S). Finally, we found that either DHA or MTX suppressed the phosphorylation of IKKα and P65 in the skin tissue (Figure 3G, H), indicating that both DHA and MTX downregulate NFκB signaling in skin.

### DHA largely prevents the recurrence of psoriatic skin lesions induced by secondary but lower doses of IMQ treatment

**Figure 4:**
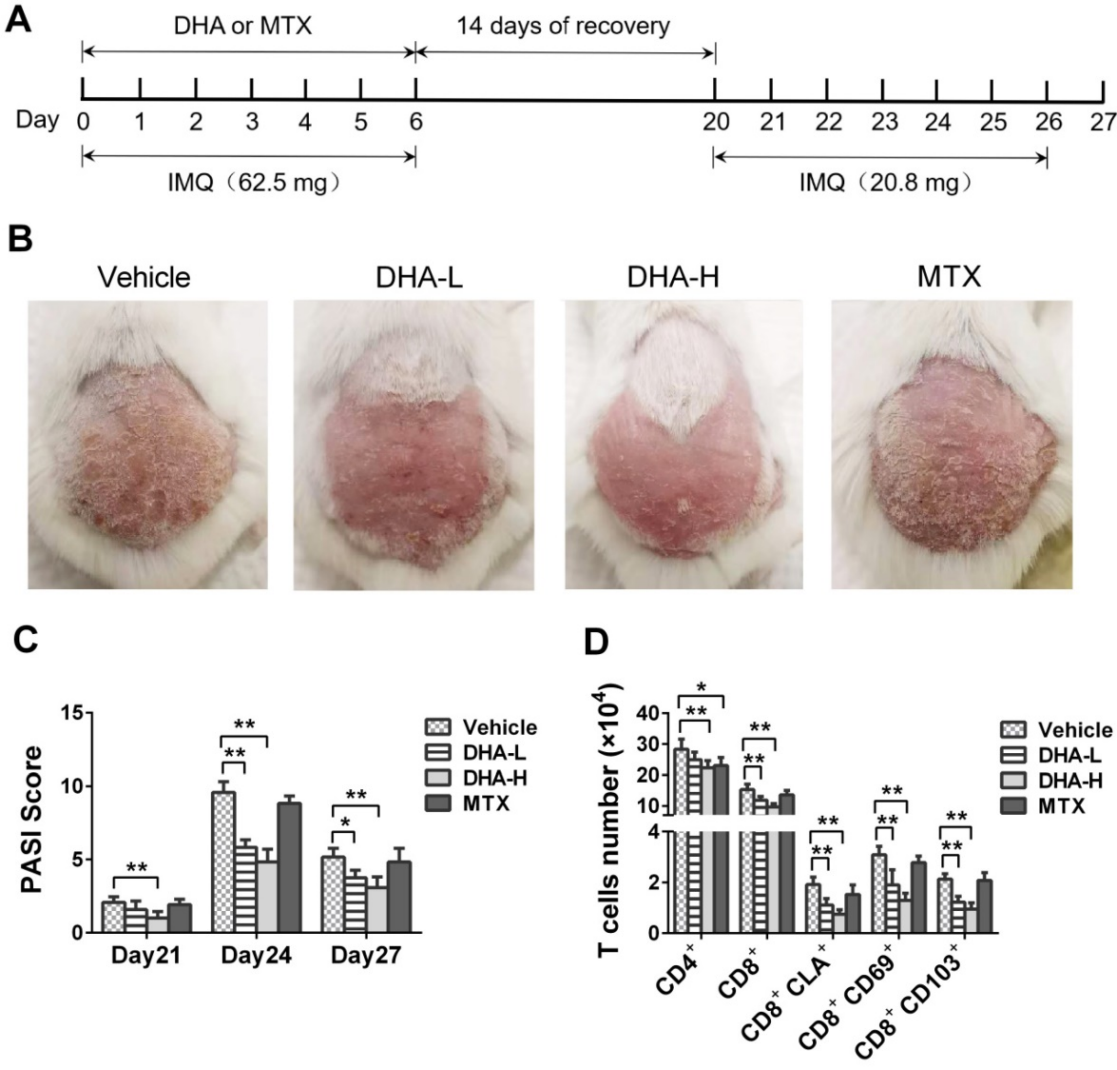
** DHA ameliorates the recurrence of skin lesion of IMQ-induced psoriatic mice and reduces skin-infiltrating CD8+ T cells**. (**A**) The timeline of the treatment. BALB/c mice were intraperitoneally administered with DHA (25 mg/kg or 50 mg/kg) or MTX for 7 consecutive days during the first application of IMQ (62.5 mg). After 14 days of recovery, all mice were re-challenged with secondary application of smaller doses of IMQ (20.8 mg) for 7 more days. (**B**) The representative photos of dorsal skin lesion of mice on day 24 (peak time for the lesion). (**C**) The PASI scores of the skin lesion in the psoriatic mice on days 21, 24 and 27. (**D**) The number of T cells, including total CD4^+^, CD8^+^ T cells and CD8^+^ T_RM_ cells, in the skin was measured via FACS analysis on day 27. All data are presented as the mean ± SD from three separate experiments (n = 6 mice/group, *p < 0.05 and **p < 0.01).

Mice were treated with IMQ without or with DHA/MTX for 7 days, rested for 14 days and then re-challenged with low doses of IMQ without DHA for 7 days, as depicted in Figure 4A. The representative images of skin lesions in all groups were shown in Figure 4B. On day 4 from secondary IMQ challenging (day 24 from the beginning of initial IMQ priming), mice of Vehicle or MTX group showed severe psoriasiform skin lesions despite being given only one-third dose of IMQ after 14 days of recovery. However, mice previously treated with DHA showed only mild skin lesions (Figure 4B). Moreover, PASI scores of DHA-treated mice were significantly decreased compared to those of Vehicle group on days 21, 24 and 27, respectively (Figure 4C). However, there was no significant difference in PASI scores between Vehicle and MTX-treated groups of mice on days 21, 24 and 27, respectively (Figure 4C). On the other hand, we enumerated T cells in the skin via FACS analysis and found that MTX reduced total number of CD4^+^, but not CD8^+^, T cells while DHA mainly decreased CD8^+^ T cells at either low or high doses, but lowered CD4^+^ cell number only at high doses (Figure 4D). More importantly, DHA, but not MTX, significantly reduced CD8^+^ T_RM_ cells, including CD8^+^CLA^+^, CD8^+^CD69^+^ and CD8^+^CD103^+^ cells, in the skin of psoriatic mice after mice were re-challenged with IMQ (Figure 4D). These results indicate that DHA, but not MTX, alleviates the recurrence of psoriatic skin lesion while diminishing CD8^+^ T_RM_ cells as well.

### DHA lowers frequency of CD8^+^, but not CD4^+^, T_CM_ cells in the psoriatic mice

**Figure 5:**
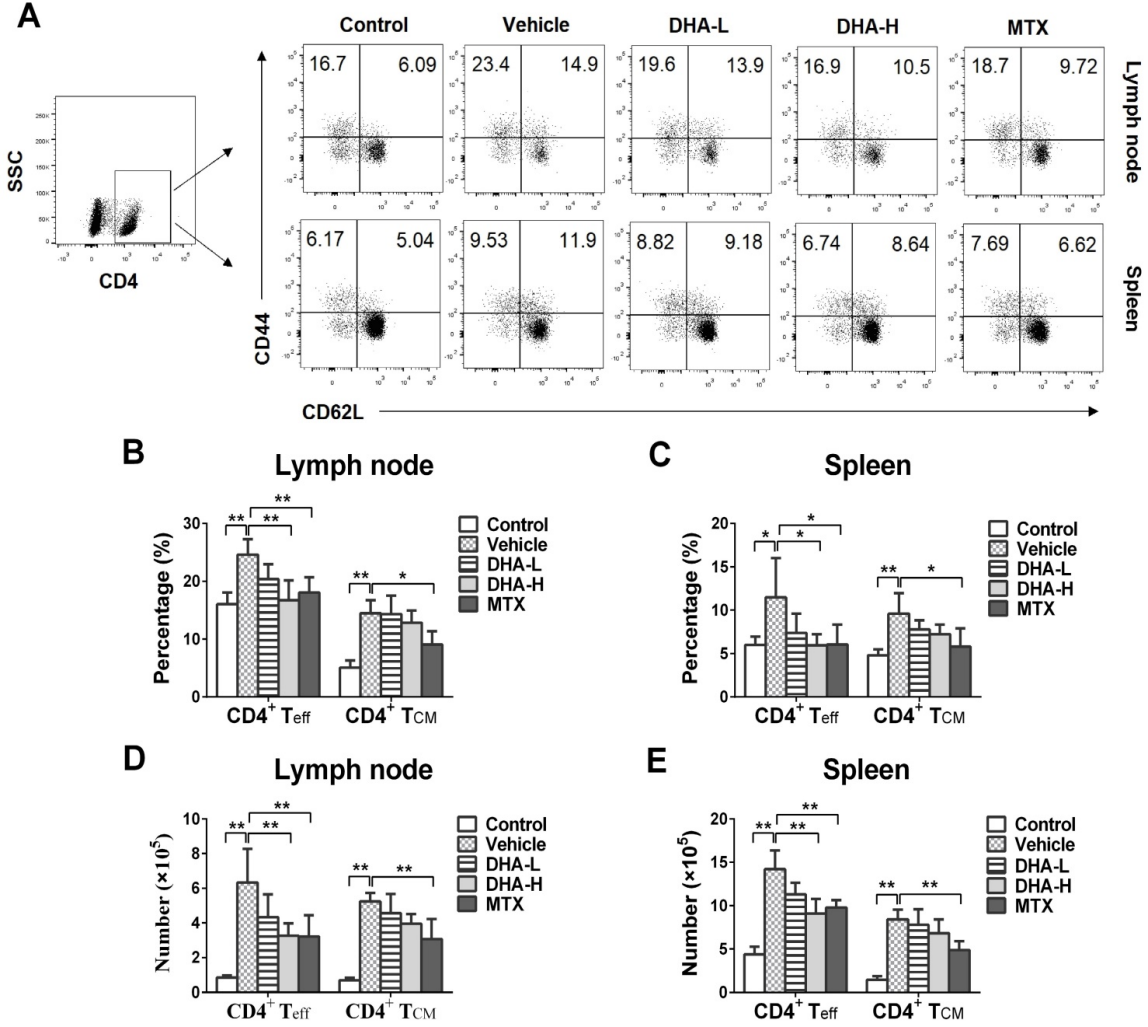
** DHA reduces the frequency of CD4^+^ effector T (T_eff_), but not CD4^+^ central memory T (T_CM_), cells in IMQ-induced psoriasis-like mice.** Lymph node and spleen cells were isolated from psoriatic mice on day 7 following various treatments. The percentages of CD4^+^ T_eff_ cells (CD4^+^CD44^+^CD62L^-^) and CD4^+^ T_CM_ cells (CD4^+^CD44^+^CD62L^+^) in lymph nodes and spleen of the psoriatic mice were determined via FACS. Shown on the top are representative dot plots of CD4^+^ T_eff_ and CD4^+^ T_CM_ cell populations in lymph nodes and spleen of the mice (**A**). Column graphs display the percentages (**B-C**) or absolute numbers (**D-E**) of CD4^+^ Teff and CD4+ T_CM_ cells in lymph nodes and spleen of the mice. Data of column graphs are presented as the mean ± SD from three separate experiments (n = 4-6 mice/group, *p < 0.05 and **p < 0.01).

**Figure 6:**
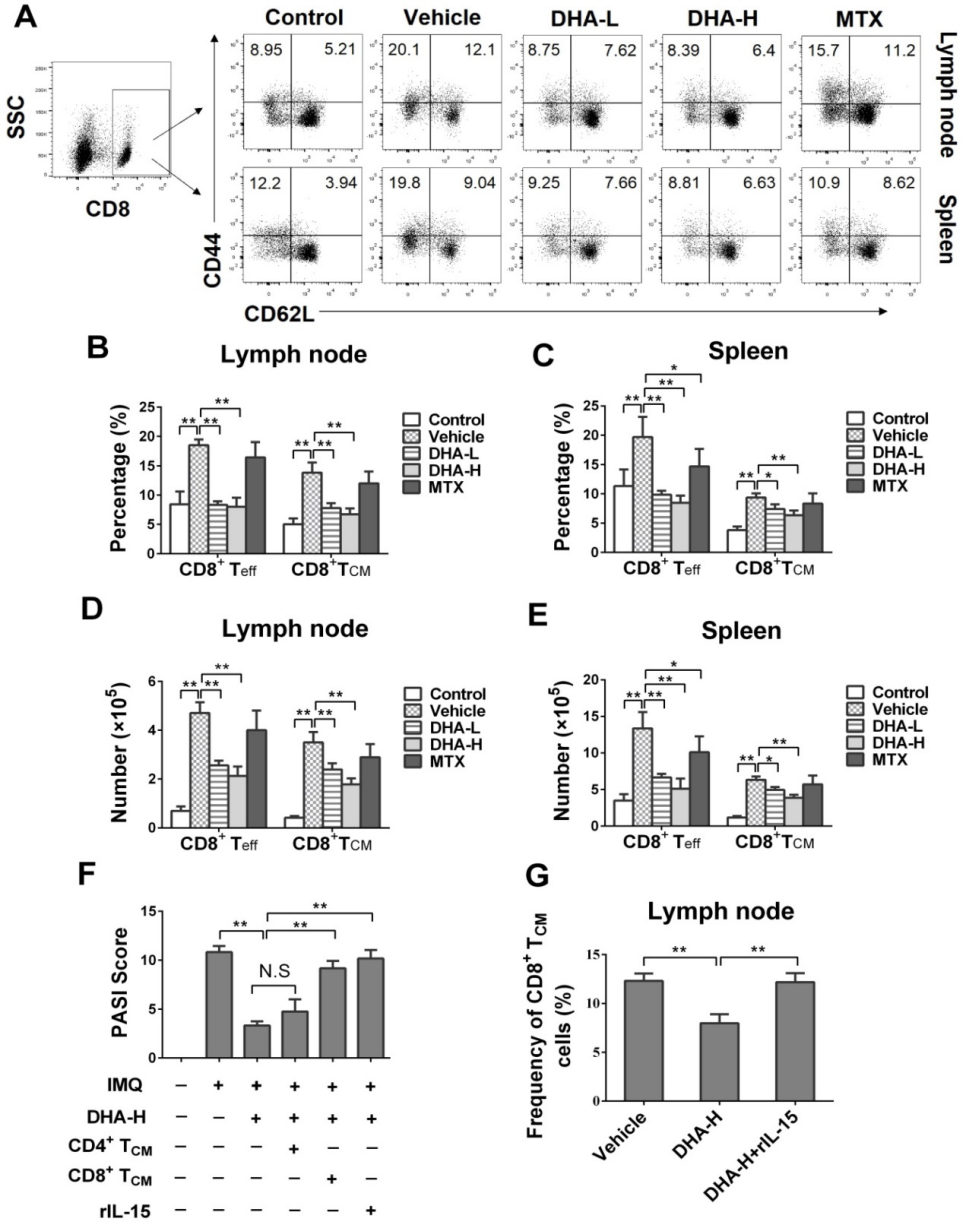
** DHA decreases the frequency of CD8^+^ effector T (T_eff_) cells and CD8^+^ central memory T (T_CM_) cells in IMQ-induced psoriasis-like mice.** Lymph node and spleen cells were isolated from psoriatic mice on day 7 following various treatments. The percentages of CD8^+^ T_eff_ cells (CD8^+^CD44^+^CD62L^-^) and CD8^+^ T_CM_ cells (CD8^+^CD44^+^CD62L^+^) in lymph nodes and spleen of the psoriatic mice were determined via FACS. (**A**) Shown are representative dot plots of CD8^+^ T_eff_ and CD8^+^ T_CM_ cell populations in lymph nodes and spleen. The percentages (**B-C**) or absolute numbers (**D-E**) of CD8^+^ T_eff_ and CD8^+^ T_CM_ cells in lymph nodes and spleen of mice were also determined. (**F**) On day 24, PASI scores of the skin lesion in psoriatic mice treated with DHA (50 mg/kg) plus administration of CD4^+^ T_CM_ cells, CD8^+^ T_CM_ cells or rIL-15 were observed. Briefly, mice received FACS-sorted CD4^+^ or CD8^+^ T_CM_ cells on day 20, the beginning day of IMQ re-challenging, while others were administered with rIL-15 on days 0, 3 and 6 during initial IMQ priming and DHA treatment. (**G**) The percentages of CD8^+^ T_CM_ cells in lymph nodes of psoriatic mice treated without or with DHA (50 mg/kg) plus rIL-15. All data of column graphs are presented as the mean ± SD from three separate experiments (n = 5-6 mice/group, *p < 0.05 and **p < 0.01).

To evaluate the effects of DHA on effector T (T_eff_) and T_CM_ cells, lymph node and spleen cells were isolated on day 7 following initial treatments and then stained to quantify T_eff_ and T_CM_ cells *via* FACS. As shown in Figure 5A-E, IMQ treatment (Vehicle group) significantly increased the percentage and absolute number of CD4^+^CD44^+^CD62L^-^ effector T (CD4^+^ T_eff_) cells and CD4^+^CD44^+^CD62L^+^ central memory T (CD4^+^ T_CM_) cells in lymph nodes and spleen of the mice compared with normal control group, while MTX largely reversed these effects of IMQ. However, DHA, even at high doses, only reduced the frequency and absolute number of CD4^+^ T_eff_, but not CD4^+^ T_CM_, cells in the lymph nodes and spleen. In contrast, DHA decreased the percentage and absolute number of CD8^+^CD44^+^CD62L^-^ effector T (CD8^+^ T_eff_) and CD8^+^ T_CM_ cells in both lymph nodes and spleen of psoriatic mice (Figure 6A-E). However, MTX only lowered the frequency and absolute number of CD8^+^ T_eff_ cells in the spleen of the psoriatic mice, but did not alter the percentage and number of CD8^+^ T_CM_ cells. Similar results regarding effects of DHA on T_CM_ frequency were also observed at the time point of day 20 (data not shown). Thus, DHA indeed shrinks CD8^+^ T_CM_ pool while reducing also CD8^+^ T_eff_ cells.

### Reconstitution of CD8^+^, but not CD4^+^, T_CM_ cells reversed the therapeutic effects of DHA on psoriasis relapse

To further confirm the role of CD8^+^ T_CM_ cells in psoriasis relapse and identify target cells of DHA, DHA-treated mice received exogenous CD4^+^ or CD8^+^ T_CM_ cells on day 20, the beginning date of IMQ re-challenging, while others were administered with rIL-15 on days 0, 3 and 6 during initial IMQ priming, as depicted in Figure 4A. As shown in Figure 6F, we found that infusion of CD8^+^ T_CM_ cells mostly reversed DHA-mediated reduction in the PASI scores on day 24, the peak time for skin lesions, whereas injection of CD4^+^ T_CM_ cells failed do so. In addition, we demonstrated that administration of recombinant IL-15 also reversed the effects of DHA on PASI scores because IL-15 significantly increased the frequency of CD8^+^ T_CM_ cells of the psoriatic mice (Figure 6G).

### DHA reduces EOMES and BCL-6 expression in CD8^+^ T cells of psoriatic mice

**Figure 7:**
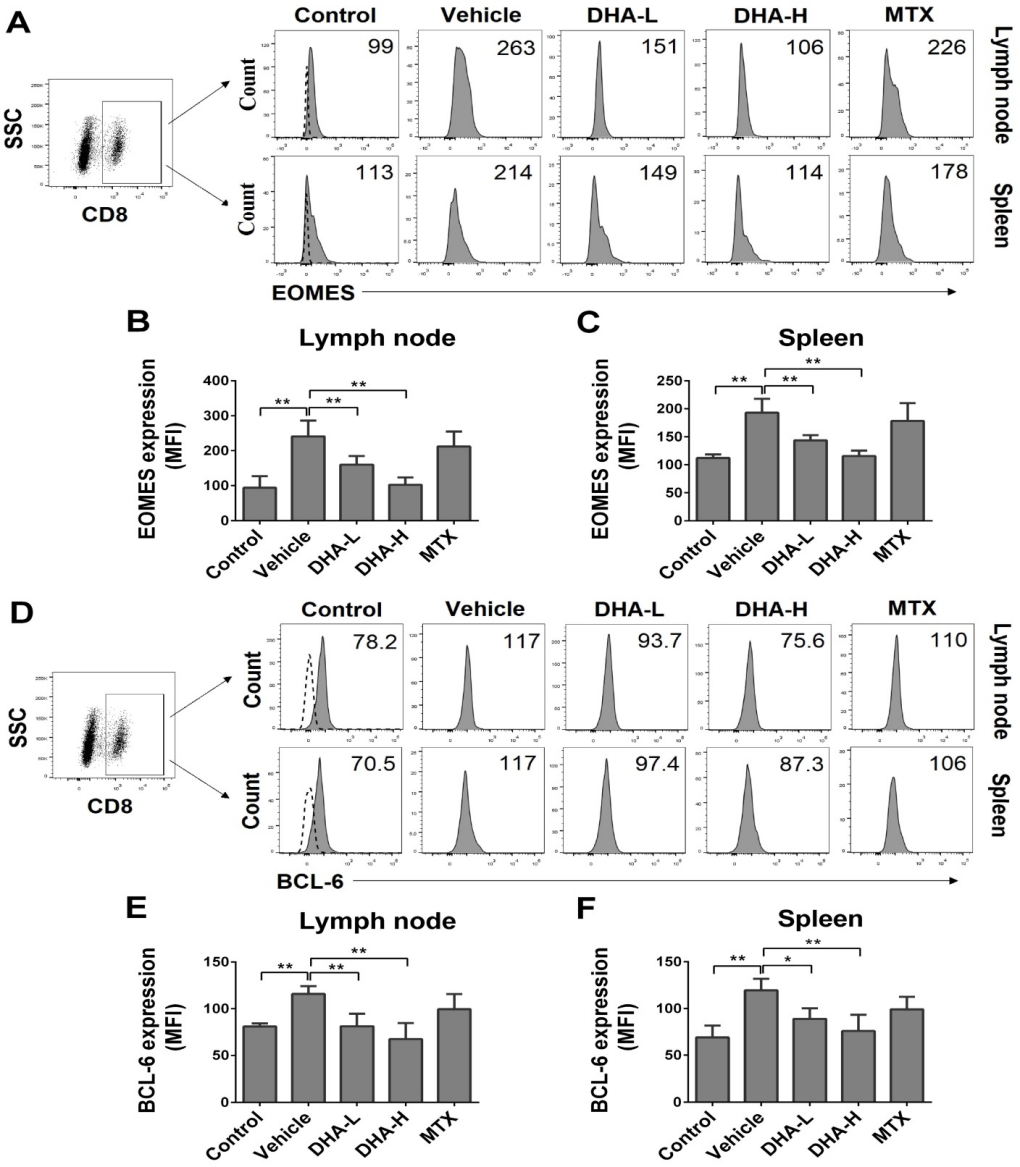
** DHA inhibits EOMES and BCL-6 expression in CD8^+^ T cells of IMQ-induced psoriasis-like mice.** Lymph node and spleen cells were isolated from psoriatic mice after 7 days of initial treatments. Expression of EOMES and BCL-6 by CD8^+^ T cells in lymph nodes and spleen of mice was determined by FACS following intracellular staining. (**A**) Representative histogram graphs of EOMES expression in CD8^+^ T cells. (**B-C**) Column graphs show the MFI of EOMES in CD8^+^ T cells from lymph nodes and spleen of the mice. (**D**) Representative histogram graphs of BCL-6 expression in CD8^+^ T cells. (**E-F**) Column graphs show the MFI of BCL-6 in CD8^+^ T cells from lymph nodes and spleen of the mice. Dotted line represents the corresponding isotype controls. Data are presented as the mean ± SD from three separate experiments (n = 4-6 mice/group, *p < 0.05 and **p < 0.01).

EOMES and BCL-6 are key transcription factors for T_CM_ development as they facilitate T cell differentiation into memory 43. Since we found that DHA lowered the frequency of CD8^+^ T_CM_ cells in psoriatic mice, we asked whether DHA regulated the expression of EOMES and BCL-6 in CD8^+^ T cells. As shown in Figure 7A-C, the expression of EOMES in CD8^+^ T cells was significantly decreased in both lymph nodes and spleen when mice were treated with DHA. Similarly, DHA also significantly reduced the expression of BCL-6 in CD8^+^ T cells in psoriatic mice (Figure 7D-F). These results indicate that DHA reduces the frequency of CD8^+^ T_CM_ cells by inhibiting EOMES and BCL-6 expression in CD8^+^ T cells. However, MTX did not alter their expression of EOMES and BCL-6.

### DHA decreases the frequency of CD8^+^ T_RM_ cells in the skin of the psoriatic mice

**Figure 8:**
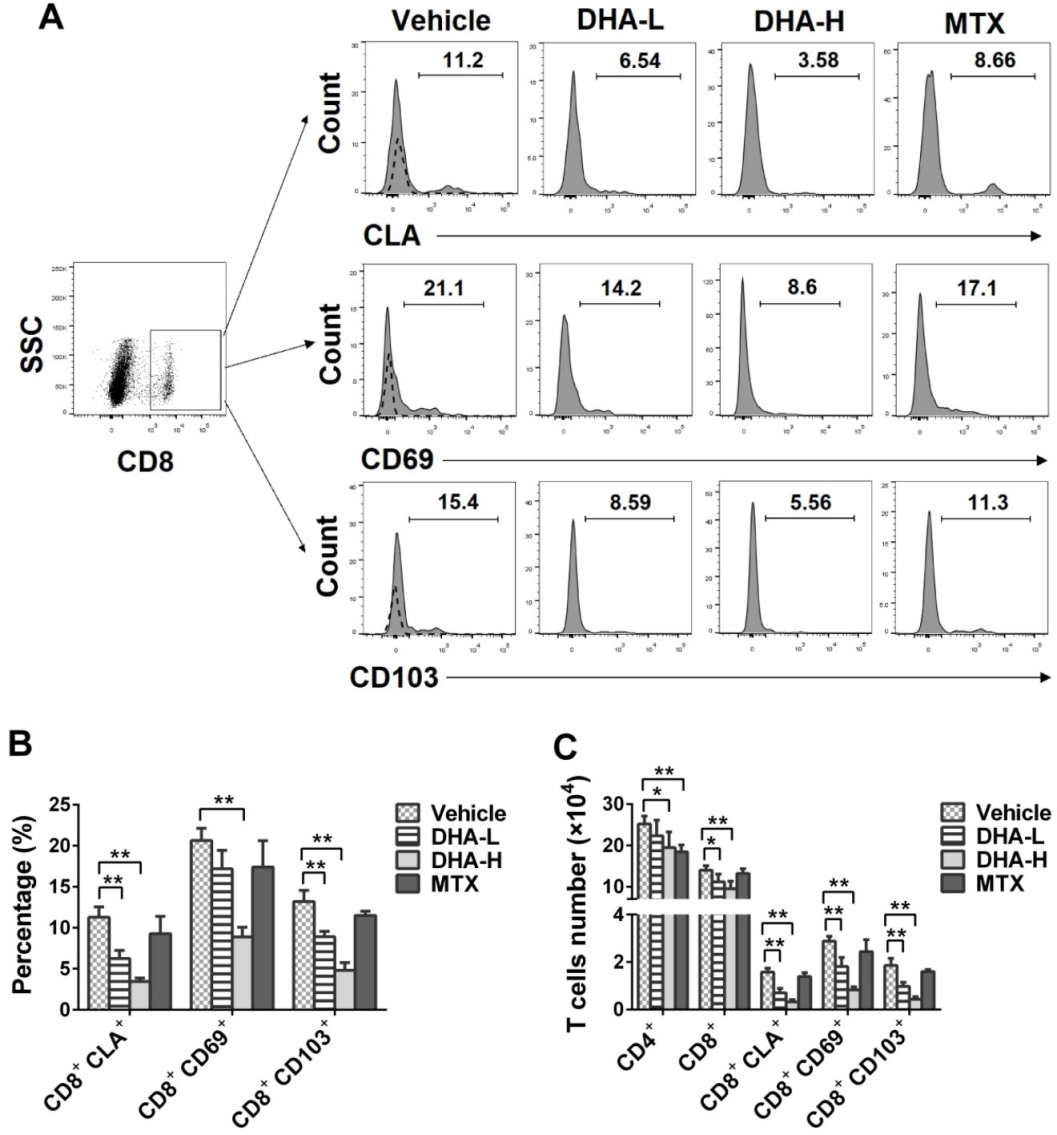
** DHA decreases the frequency of CD8^+^CLA^+^, CD8^+^CD69^+^ and CD8^+^CD103^+^ T_RM_ cells in the skin of IMQ-induced psoriatic mice.** Skin-infiltrating cells were isolated from psoriatic mice on day 7. The percentages of CLA^+^, CD69^+^ or CD103^+^ cells within CD8^+^ T cell subset were determined *via* FACS analysis. (**A**) Representative histograms of CLA^+^, CD69^+^ or CD103^+^ cells after gating on CD8^+^ T cell subset. (**B**) Column graphs show the percentages of CLA^+^, CD69^+^ or CD103^+^ cells within CD8^+^ T cell population. (**C**) Shown are the absolute numbers of CD8^+^CLA^+^, CD8^+^CD69^+^ and CD8^+^CD103^+^ T_RM_ cells as well as total CD4^+^/CD8^+^ T cells. Dotted line represents the corresponding isotype controls. Data are presented as the mean ± SD from three separate experiments (n = 4-6 mice/group, *p < 0.05 and **p < 0.01).

It has been previously reported that CD69, CD103 and CLA are highly expressed on the surface of T_RM_ cells in the skin of patients with psoriasis 14. On day 7, we isolated skin-infiltrating cells and determined the percentage of CLA^+^, CD69^+^ or CD103^+^ cells within CD8^+^ T-cell subset in the skin of the psoriatic mice *via* FACS analysis. As shown in Figure 8A-C, the percentage and absolute number of CD8^+^CLA^+^, CD8^+^CD69^+^ or CD8^+^CD103^+^ T_RM_ cells were significantly decreased when the psoriatic mice were treated with DHA, especially at high doses, whereas MTX failed to do so. Moreover, DHA lowered total CD8^+^ T-cell number in the skin whereas MTX reduced CD4^+^, but not CD8^+^, T-cell number (Figure 8C). These results indicate that DHA, but not MTX, inhibits the development of CD8^+^ T_RM_ cells in the skin of psoriatic mice.

### DHA alleviates human skin lesion in humanized mice grafted with lesional skin from psoriatic patients

**Figure 9:**
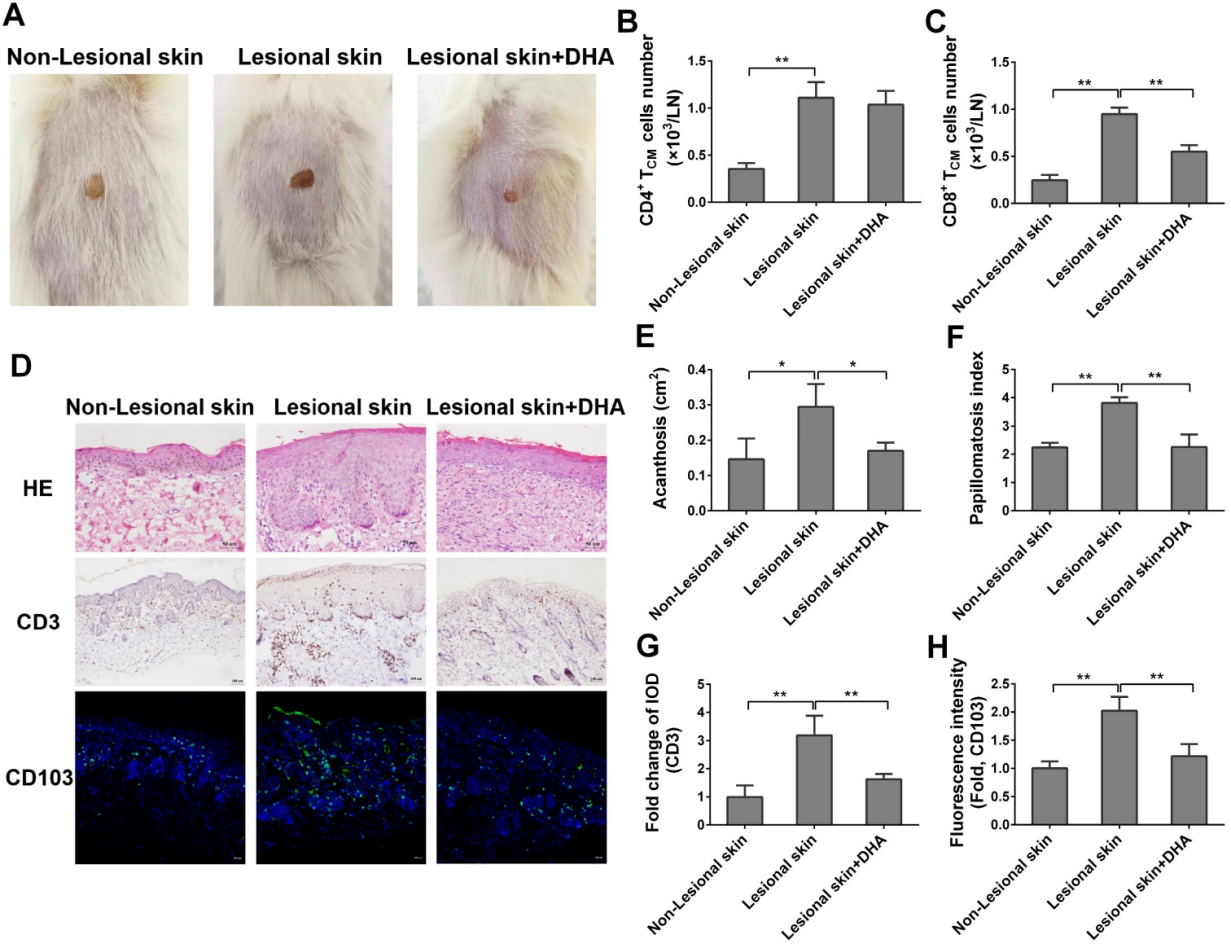
** DHA alleviates human skin lesion in humanized mice grafted with lesional skin from psoriatic patients.** The lesional or non-lesional skin from psoriatic patients was transplanted to the dorsal flank area of NSG mice that received PBMCs from the same patient. Mice were then treated with DHA at doses of 50 mg/kg/day. (**A**) Shown are the representatives of lesional and non-lesional human skin grafts in NSG mice with or without DHA treatment 8 days after transplantation. (**B-C**) Human CD4^+^ or CD8^+^ T_CM_ cells (CD45RO^+^CCR7^+^) from draining LNs of humanized NSG mice that received both psoriatic human skin and PBMCs were enumerated by FACS on day 8. (**D**) Representative images of H&E and CD3 stainings of the grafted lesional and non-lesional human skin as well as immunofluorescence staining of CD103 on day 8 (Scale bar, 50 or 100 µm). (**E-F**) Acanthosis and papillomatosis index of skin based on H&E staining above. (**G**) Fold changes in CD3 expression (IOD) in the grafted lesional and non-lesional skin. (**H**) The Fluorescence intensity of CD103 in the skin grafts based on immunofluorescence staining. Data are presented as the mean ± SD of four recipient mice (n = 4 mice/group).

To further explore the potentially therapeutic effects of DHA on human psoriasis, we transplanted the lesional or non-lesional skin from psoriatic patients to the dorsal flank area of NSG mice that received PBMCs from the same patients. Mice were then treated with DHA at a dose of 50 mg/kg/day. There was no any clear sign of GVHD in our humanized NSG mouse model within the first 8 days. As shown in Figure 9A, the lesional skin grafts in NSG recipient mice exhibited continuous psoriatic skin lesion 8 days after transplantation compared to non-lesional skin grafts. However, the lesion of originally lesional skin grafts in NSG recipient mice was dramatically attenuated when the mice were treated with DHA, indicating that DHA alleviates psoriatic human skin inflammation in recipient mice. Moreover, we determined the absolute cell number of human T_CM_ cells in draining LNs of NSG mice by FACS on day 8. As shown in Figure 9B-C, we found that DHA significantly reduced the cell number of human CD8^+^ T_CM_ cells (CD8^+^CD45RO^+^CCR7^+^), but not CD4^+^ T_CM_ cells (CD4^+^CD45RO^+^CCR7^+^). This result was consistent with the finding of murine T_CM_ cells in psoriatic wild-type mice. Furthermore, H&E staining revealed that DHA significantly alleviated the acanthosis and papillomatosis index of grafted lesional human skin (Figure 9D-F). We also found that DHA significantly inhibited CD3 expression in the skin (Figure 9D & 9G). Finally, immunofluorescence staining showed that the expression of CD103 in grafted lesional skin was downregulated by DHA, suggesting that DHA also reduces the presence of CD103+ T_RM_ cells in human skin graft derived from psoriatic patients (Figure 9D & 9H).

## Discussion

Psoriasis is a chronic inflammatory skin disease that is prone to recurrence. Systemic immunosuppression, including cyclosporine, MTX and other biological agents, can alleviate psoriasis. However, the skin lesions may recur in many psoriatic patients once they stop taking these drugs. Further, conventional immunosuppressive agents may cause various side effects. Thus, developing new drugs for treating psoriasis are always needed to prevent its recurrence. DHA, a derivative of artemisinin, has been demonstrated to have immunosuppressive effects on some inflammatory diseases, such as encephalomyelitis, thyroiditis and lupus nephritis, in experimental animal models 32, 34, 37 although it remains unknown whether DHA exerts an effect on these diseases in clinic. Moreover, DHA can regulate the differentiation and apoptosis of T cells 23, 32. However, it is unknown whether DHA has a therapeutic effect on psoriasis. In this study, we have provided the first evidence that DHA ameliorates IMQ-induced psoriasiform skin inflammation in both wild-type and humanized mice and alleviates its recurrence as well.

Although the pathogenesis of psoriasis recurrence remains unclear, it is assumed that memory T cells play a critical role in its relapse. When naïve T cells in psoriasis patients are activated, they proliferate and transform into effector T cells and memory T cell precursors 44. These memory precursors then develop into several subsets of memory T cells, including effector memory T (T_EM_), T_CM_ and T_RM_ cells 14, 45, 46. T_RM_ cells reside in the skin of psoriatic patients for long-term while T_CM_ cells can egress from lymph nodes to blood or tissue and become effector T cells upon re-encounter with antigens 10, 47. T_CM_ cells may migrate to skin and give rise to T_RM_ cells 45, 46. Memory T cells are also somewhat resistant to immunosuppression 48 or immunoregulation 49. Few studies have demonstrated that CD4^+^ T_CM_ cells are significantly increased in patients with psoriasis 10 while circulating CLA^+^CCR4^+^CD8^+^ T_CM_ cells are also expanded in the patients 11, indicating that T_CM_ cells may be involved in the pathogenesis of psoriasis. On the other hand, T_RM_ cells never recirculate through the blood once they reside in the skin. Most of them in skin lesions express CLA, CD103 and CD69 17, 19. Many studies have shown that T_RM_ cells participate in the pathogenesis of psoriasis. It has been reported that T_RM_ cells producing IL-17 are enriched in both non-lesional skin and resolved lesional skin of psoriasis patients, leading to the recurrence of psoriasis in the same region 15, 16, 50. Particularly, CD8^+^ T_RM_ cells have also been proved to produce IL-17 and to be associated with clinical progression of psoriasis 7, 17, 51. Indeed, we found that DHA suppressed IL-17A expression in the psoriasiform skin while reducing CD8^+^ T_RM_/T_CM_ cells. Thus, DHA may attenuate the recurrence of psoriasis by suppressing CD8^+^ T_RM_/T_CM_ cells. However, it remains to be defined if a reduction in CD8^+^ T_RM_ cells by DHA is caused by its first suppression of CD8^+^ T_CM_ cells. It's possible that CD8^+^ T_RM_, but not T_CM_, cells directly mediate the recurrence of psoriasis while CD8^+^ T_CM_ cells may act as a precursor sustaining CD8^+^ T_RM_ cells.

In the present study, we revealed that the frequency of CD8^+^ T_CM_ cells was dramatically increased in the psoriatic mice while DHA reduced their frequency. Interestingly, DHA did not significantly shrink CD4^+^ T-cell memory pool. In contrast, we demonstrated that MTX, one of the effective traditional drugs for the treatment of psoriasis, lowered the frequency of CD4^+^, but not CD8^+^, T_CM_ cells. The mechanisms underlying this striking difference between DHA and MTX remain to be defined. Since we found that DHA attenuated the recurrence of psoriasis and diminished CD8^+^, but not CD4^+^, T_CM_ pool, it's possible that CD8^+^ T_CM_ cells play a more important role in psoriasis relapse than do CD4^+^ T_CM_ cells. Indeed, adoptive transfer of CD8^+^, but not CD4^+^, T_CM_ cells to DHA-treated psoriatic mice reversed the therapeutic effects of DHA on psoriasis relapse. Therefore, DHA could hold an advantage over MTX in preventing the recurrence of psoriasis. On the other hand, we revealed that DHA decreased the frequency of CLA^+^, CD69^+^ or CD103^+^ T_RM_ cells within CD8^+^ T-cell subset in the skin of psoriatic mice while MTX failed to do so, indicating that DHA is also more effective in suppressing CD8^+^ T_RM_ cells than MTX. Ozcan *et al.* found that nanoparticle-coupled topical MTX normalized immune responses and ameliorated psoriasis in mice 52. They found that the coupled MTX mainly reduced CD4^+^ T cell number in the skin, with CD8^+^ T cells less affected. That finding is actually consistent with our findings that MTX significantly lowered the percentage and number of CD4^+^ T cells, including both Teff and T_CM_ cell components, whereas CD8^+^ T cells were less affected by MTX since we found that MTX did not influence CD8^+^ T_CM_ or CD8^+^ T_RM_ cells, but only moderately reduced CD8^+^ Teff cells in the spleen. Finally, we demonstrated that DHA significantly reduced cell number of human CD8^+^ T_CM_ cells in humanized NSG mice that simultaneously received human skin grafts and PBMCs from the same patient. DHA also decreased the number of CD103^+^ T_RM_ cells in human skin grafted in the NSG mice. Thus, our findings may have important clinical implications for the treatment of human psoriasis since its recurrence is currently a major medical problem.

EOMES and BCL-6 are some of the key transcription factors for the formation of memory T cells, especially CD8^+^ T_CM_ 53, 54. It has been reported that EOMES-deficient CD8^+^ T cells are unable to generate self-renewing T_CM_ and defective in homeostatic turnover as well as long-term survival 55, 56. Moreover, a recent study has found that EOMES maintains the expression of CD62L in CD8^+^ T_CM_ cells, thus enhancing their trafficking to and retention in secondary lymphoid organs 57. BCL-6 is also crucial for the formation of CD8^+^ T_CM_ cells. The numbers of CD8^+^ T_CM_ cells were greatly reduced in the spleen of BCL-6-deficient mice, whereas their proliferation upon secondary stimulation dramatically accelerated in BCL-6 transgenic mice 58. In our study, we found that DHA decreased the expression of both EOMES and BCL-6 in CD8^+^ T cells. Thus, the inhibitory effects of DHA on CD8^+^ T_CM_ formation may be attributed to its inhibition of EOMES and BCL-6 expression in CD8^+^ T cells. However, it cannot be ruled out that a decrease in EOMES and BCL-6 expression in total CD8^+^ T cells could be ascribed to the reduction of the percentage of CD8^+^ T_CM_ cells in our experimental setting.

Previous studies have shown that IL-15 plays an important role in CD8^+^ T cell memory generation 59. Blocking IL-15 biological activity reportedly reduced the severity of psoriasis in a xenograft model of human psoriasis 60. Since our data demonstrated that DHA inhibited IL15 expression, it's likely that DHA reduced CD8^+^ T_CM_ cells by suppressing IL-15 expression. Interestingly, Bouchaud *et al.* have reported that soluble IL-15 Rα produced by keratinocytes in the skin is an endogenous antagonist of psoriasiform skin inflammation 61 whereas CD8^+^CD122^low^ memory T cells are independent of IL-15 62, indicating that there are IL-15-independent mechanisms underlying the homeostasis of CD8^+^ memory T cells. We found that DHA decreased IL-15 expression but did not significantly alter IL-15 Rα level in the skin.

## Conclusions

DHA, a derivative of Nobel Prize-winning artemisinin family, not only is as effective as MTX, a conventional immunosuppressive agent, in ameliorating the acute skin inflammation of psoriasis, but also is better than MTX in alleviating the recurrence of psoriasis via reducing CD8^+^, but not CD4^+^, T cell memory, including both CD8^+^ T_CM_ and CD8^+^ T_RM_ cells. Therefore, our study has provided the first evidence that DHA may be a promising drug for treating human psoriasis and its recurrence. Further studies are warranted to determine its safety or toxicity before its clinical usage.

## Supplementary Material

Supplementary figures and tables.

## Abbreviations

DHA: dihydroartemisinin
EOMES: eomesodermin
IMQ: imiquimod
MTX: methotrexate
PASI: psoriasis area and severity index
T_CM_: central memory T cell
T_eff_: effector T cell
T_RM_: resident memory T cell
