# Supplementary material for: Dihydroartemisinin ameliorates psoriatic skin inflammation and its relapse by diminishing CD8+ T-cell memory in wild-type and humanized mice
Source: Theranostics. 2020 Aug 21;10(23):10466–82. doi: 10.7150/thno.45211 (PMC7482806; doi:10.7150/thno.45211)
Supplement: Supplementary file 1 — Supplementary figures and tables. [file thnov10p10466s1.pdf]

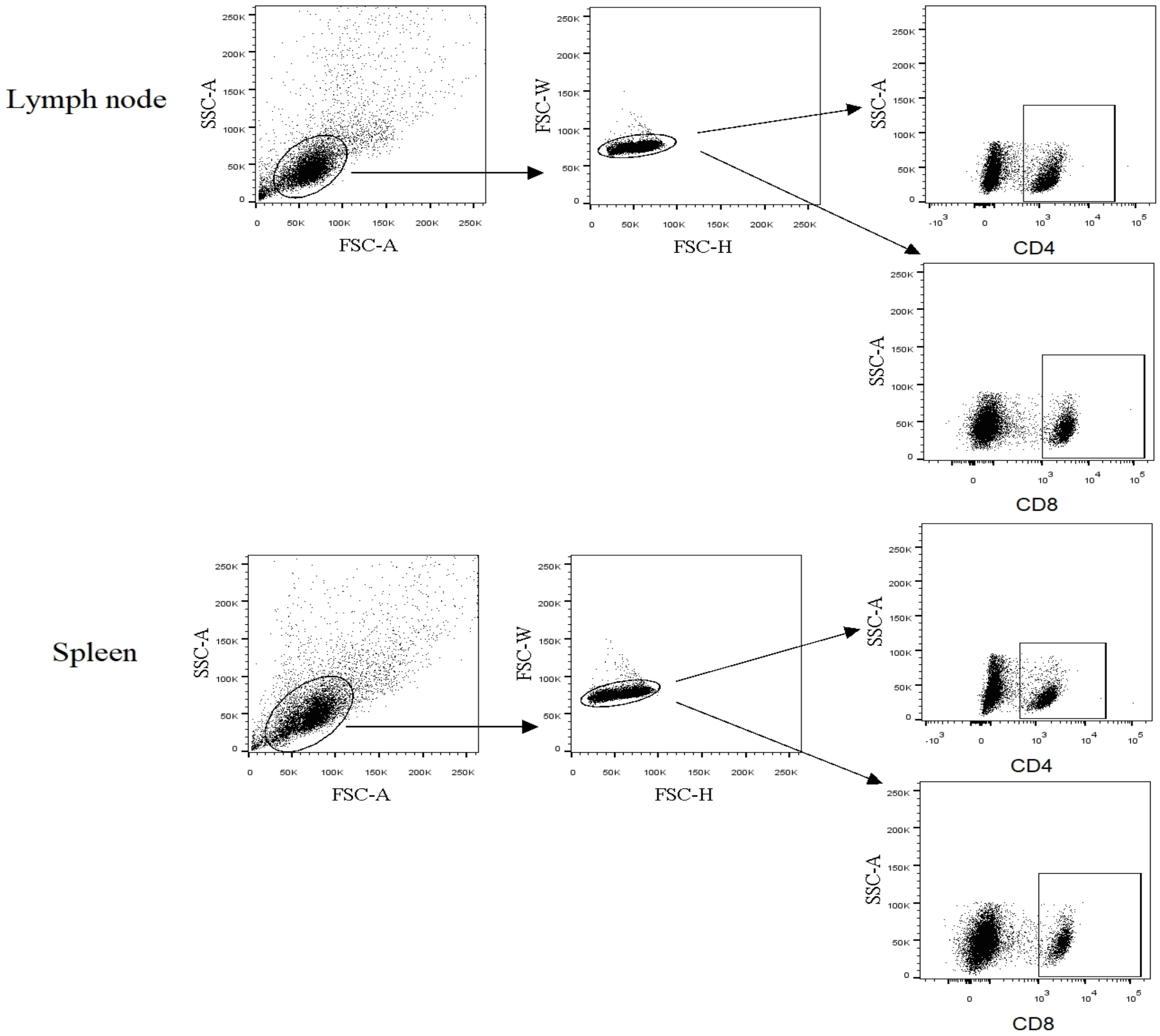

Supplementary Figure 1S: **Gating strategy**

Shown above are gating strategies for spleen and lymph node cells derived from IMQ-induced psoriatic mice for further FACS analysis of CD4+ or CD8+ T cells.

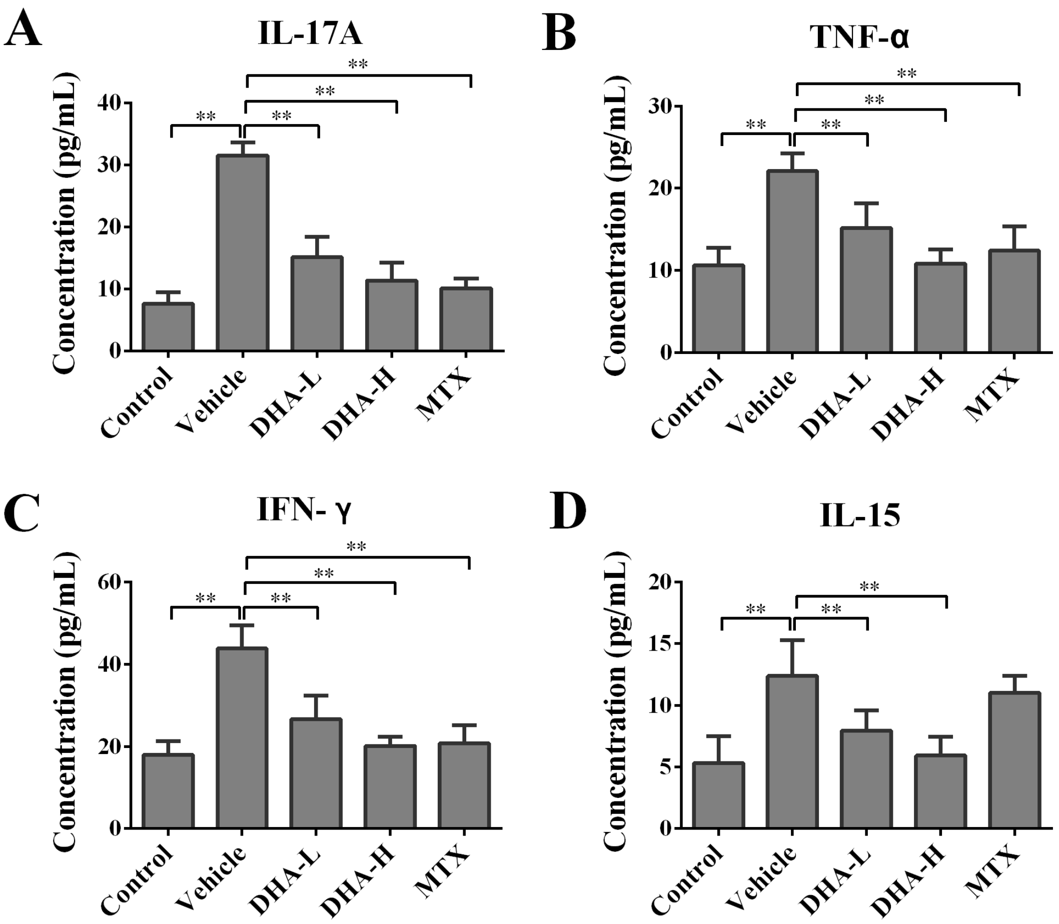

Supplementary Figure 2S: DHA reduces protein level of IL-15 and IL-17 in the serum of IMQ-induced psoriatic mice.

Protein level of IL-15, IL-17A, IFNγ and TNFα in the serum of the IMQ-induced psoriatic mice was determined via ELISA after 7 days of the treatment with either dihydroartemisinin (DHA) or methotrexate (MTX). Data of column graphs are presented as the mean ± SD from three separate experiments (n=5-6 mice/group, **$P < 0.01$). DHA lowered the protein level of IL-15, IFNγ and TNFα in the serum of IMQ-induced psoriatic mice.

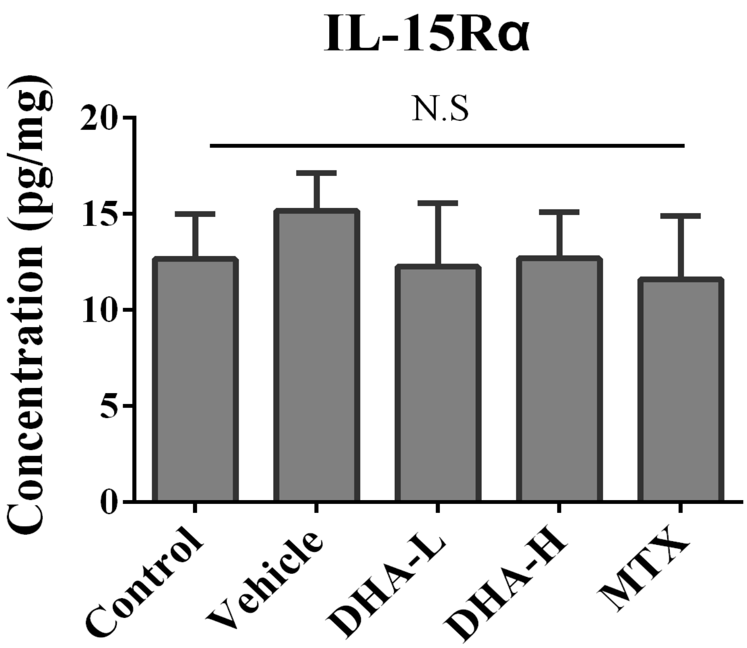

Supplementary Figure 3S: DHA does not significantly alter IL15Rα level in the skin of IMQ-induced psoriatic mice.

IL-15Rα level in the protein solution derived from skin tissue of the IMQ-induced psoriatic mice was determined via ELISA after 7 days of treatment with either DHA MTX. Data of column graphs are presented as the mean ± SD from three separate experiments (n=6 mice/group, N.S: Non-significant).
